# The impact of imaging time and contrast agent dose on screening for osteoporosis with contrast-enhanced CT

**DOI:** 10.1186/s41747-021-00259-5

**Published:** 2022-02-10

**Authors:** Mischa Woisetschläger, Eva Klintström, Anna Spångeus

**Affiliations:** 1 Department of Radiology in Linköping, and Department of Health, Medicine and Caring Sciences, Linköping University, 58185 Linköping, Sweden; 2 Center for Medical Image Science and Visualization (CMIV), Linköping University, Linköping, Sweden; 3 Department of Acute Internal Medicine and Geriatrics, Department of Endocrinology and Department of Health, Medicine and Caring Sciences, Linköping University, Linköping, Sweden

**Keywords:** Bone density, Contrast media, Four-dimensional computed tomography, Osteoporosis

## Abstract

**Background:**

Screening for osteoporosis with contrast-enhanced computed tomography (CT) is promising for identifying high-risk osteoporotic patients. Our aims were (1) to investigate the estimated volume bone mineral density (vBMD) change over time after contrast injection (CT perfusion imaging, CTPI); and (2) to examine the influence of contrast dose on vBMD.

**Methods:**

Fifteen patients, aged 71 ± 9 years (mean ± standard deviation, range 55–86) underwent a CTPI examination (28 scans within 63 s) of the upper body followed (after a waiting time of 10 min) by a full 4-phase CT examination (4 scans within 4 min). The contrast dose for CTPI was 0.38–0.83 mL/kg, and for 4-phase CT was 0.87–1.29 mL/kg. Vertebrae L1–L3 were analysed totalling 43 vertebrae, using Mindways qCT Pro.

**Results:**

After contrast injection, vBMD showed a near-horizontal line until 17.5 s (non-contrast phase), followed by a steep increase 17.5–41.5 s after contrast injection, *i.e.*, in the arterial phase, which plateaued 41.5 s after, *i.e.*, in the early venous phase. A higher contrast dose per kg yielded significantly higher vBMD increase in both the arterial and venous phase (*p* < 0.003).

**Conclusions:**

Both time from contrast administration and contrast dose per kg affected vBMD results. In arterial phase, the steepness of the curve makes vBMD estimation unsure. However, as values plateaued in the venous phase it might be possible to predict the correct vBMD values. Furthermore, contrast dose is a factor that needs to be adjusted for when using such a formula.

## Key points

- Time from contrast administration affects the volume bone mineral density (vBMD) values, especially in the arterial phase.
- Contrast to weight ratio significantly affects vBMD values.
- Conversion formulas to calculate vBMD values from contrast-enhanced computed tomography scans could be calculated from venous phase scans.

## Background

Osteoporosis and its consequent increased risk of fractures go along with considerable increased morbidity and mortality [1–3] as well as a high economic burden for society with about 37 billion euros in the 27 European Union countries [4]. Patients with an osteoporotic fracture have a high risk of new fractures in near time [4–6], thus highlighting the importance of a fast and secure handling and treatment of these patients. However, despite several cost effective treatment options on the market [7], osteoporosis is still heavily underdiagnosed and undertreated [3, 8]. The reason for this is multifactorial including the risk of loss to follow-up regarding osteoporosis after fractures have been treated in the emergency setting. An organisational reason for this might be the fact that that an additional examination, *i.e.*, dual-energy x-ray absorptiometry, must be obtained before the decision of further handling of the patient regarding osteoporosis. This dual-energy x-ray absorptiometry scan requires an extra visit for the patient, and might in some settings (*e.g.*, some organisations in Sweden) include several months waiting times which further increase the risk of losing patients, and delaying treatment. Furthermore, there is often a lack of clarity regarding who is clinically responsible for this patient group, which was earlier referred by Harrington [9] as the Bermuda Triangle in osteoporosis care which is made up by the orthopaedist, primary care physician, and the osteoporosis expert into which the fracture patient disappears.

Several national and international initiatives have been initiated to ensure adequate evaluation and osteoporosis treatment after fracture. These initiatives are built on the fracture liaison services aiming to strengthen post-fracture handling of patients [10–12].

One possible solution to facilitate osteoporosis handling might be the measurement of volume bone mineral density (vBMD) from computed tomography (CT) examinations done for other indications than osteoporosis assessment. This concept has been evaluated by several groups [13–17]. Early studies used unenhanced scans with a kVp of 120. In recent years, we have seen a declining trend in the use of unenhanced scans in our department to save radiation dose. This includes the emergency examinations of the abdomen which are increasingly done after intravenous injection of contrast agent only to better answer the wider clinical referral questions, which especially in older patients often includes the suspicion of malignancy. Most of our malignancy follow-up CT scans are done with contrast only (when not contraindicated by impaired kidney function). This led to the question if vBMD can be reliably obtained from contrast-enhanced abdominal scans considering that, after intravenous injection of contrast agent, the measured vBMD values generally increase in the arterial phase and increase slightly further in the venous phase [18–21].

While several groups reported that contrast-enhanced abdominal CT examinations can be used for vBMD assessment by linear conversion formulas [19, 20, 22–25] or could be used to automatically screen patients for osteoporosis [26], other groups report variable vBMD values depending on scan delay times [27]. In addition, most of the above-mentioned studies were single-centre studies with CT protocols using kVp values fixed at 120. As an increasing number of contrast-enhanced abdominal CT scans are made with lower kVps in order to save radiation dose and contrast agents, screening methods relying on software solutions using solely internal tissue references might be problematic to use [28]. The reason for this is that the Hounsfield Units (HU) units of different tissues behave differently after contrast administration at varying kVp [29, 30].

Contrast concentrations, injection times, and speed, as well as total contrast volume differed greatly between studies. As described in the overview article by Bae [30], intravenous contrast media behaviour depends on several factors which substantially influence the HU values. These factors include the different organ sites, iodine concentration, injection duration, body weight, cardiac output, injected volume, and injection rate.

CT perfusion imaging (CTPI) is a method based on multiple CT scans during a short time (up to 1 scan/1.5 s), thus enabling to evaluate the organ timely characteristics during contrast uptake. To our knowledge, no study on vBMD analysis of CTPI data in comparison to a simultaneously derived multiphase CT is yet available.

The aims of our study were (1) to investigate the vBMD change over time after contrast injection (CTPI examination); and (2) to examine the influence of contrast dose on vBMD values.

## Methods

### Ethics and study design

**Fig. 1:**
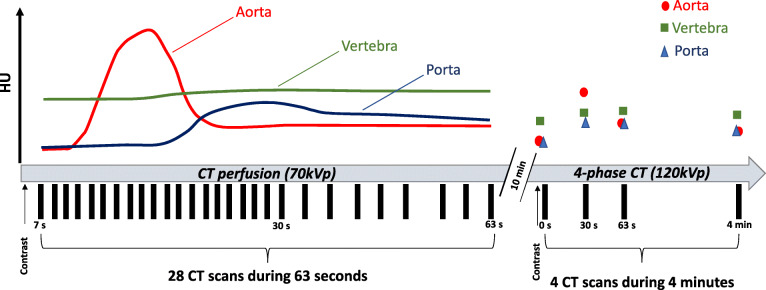
Study protocol which included: (1) a computed tomography (CT) perfusion imaging examination (28 scans performed over 63 s); (2) a 10-min break and finally; (3) a conventional 4-phase CT examination (four scans performed over 4 min). Contrast behaviour over time in three organs, *i.e.*, aorta (red), vertebra (green), and portal vein (blue) are schematically visualised. Black boxes on *x*-axis: time point of each CT scan. The HU in the aorta and portal vein were measured approximately at the height of vertebra L1. The time curves of the aorta and portal vein are given as a reference for the contrast media behaviour in different blood phases. *HU* Hounsfield units

The study was approved by regional ethics committee of the Faculty of Health Sciences, Linköping University (dnr. 2016-43/31 and dnr. 2019-05855). Informed consent was obtained from all patients. Patients from the consecutive hepatocellular carcinoma study [31] were included for a retrospective analysis of CT scans. The CT scans were performed between October 2016 to March 2019. All patients had hepatocellular carcinoma and were planned for transarterial chemoembolisation treatment. All patients first underwent a CTPI examination of the upper body (28 scans in total), and, after a waiting period of 10 min, a full 4-phase CT examination (four scans in total) of the abdomen was performed (Fig. 1). The HU units in the aorta and the portal vein were measured approximately in the height of vertebra L1. The time curves of the aorta and portal vein were considered as a reference for the contrast media behaviour in different blood phases.

### CTPI

CTPI examinations were undertaken on a Somatom Force scanner (Siemens Healthcare, Forchheim, Germany). The scanning parameters were as follows: tube voltage 70 kVp, tube current of 150 mAs with a collimation of 48 × 1.2 or 192 × 0.6 mm. The total scanning time was 62.5 s, with the first 20 scans made every 1.5 s, the following 5 scans every 3 s, and the last 3 scans (venous phase) after a waiting time of 6 s were undertaken every 3 s (Fig. 1). The first CTPI scan was taken 7 s after contrast injection, leaving the first scans with no contrast enhancement in vertebral tissue. The anatomical scan length for the first 25 scans was 22.4 cm including the upper border of the liver and including L1–L3 in most patients. The anatomical scan length for last three scans (venous phase) was 48 cm and included the whole abdomen (from the diaphragm to the symphysis). The scans were obtained with the patient moving back-and-forth through the gantry in a “pendulum” movement. Patients were instructed to take shallow breaths during imaging. A fixed dose of 50 mL of iopromide 370 mg I/kg (Ultravist™, Bayer Healthcare, Leverkusen, Germany) was injected at 6 mL/s followed by a flush of physiologic (0.9%) saline (50 mL) at 6 mL/s with a dual-head power injector (Ulrich Medical, Ulm, Germany) with a maximum inflow time of 8 s. Compression to the upper abdomen was not applied. The mean contrast to weight ratio for the CT perfusion examination was 0.62 ± 0.14 (mean ± standard deviation), ranging from 0.38 to 0.83, with a fixed amount of 50 mL of contrast agent applied.

### Four-phase CT

The same CT scanner as for the CT perfusion protocol was used to perform a 4-phase CT scan with a tube voltage of 120 kVp and a tube current of 130 mAs. The examination included an unenhanced scan and three contrast-enhanced phases, *i.e.*, arterial (30 + 2 s after contrast-injection), venous (63 + 2 s after contrast-injection), and late venous phase (4 min after contrast-injection). Low-osmolarity nonionic contrast medium iopromide 370 mg I/kg (Ultravist™, Bayer Healthcare, Leverkusen, Germany) was injected at a maximum volume of 118 mL and injection rate of 5–6 mL/s. The contrast dose per patient was calculated regarding our clinical routine with a software program called OmniVis©, version 5.1, GE Healthcare Sverige AB, Danderyd, Sweden). The iodine dose per kilogram is set to 450 mg I/kg. The mean contrast ratio for the 4-phase CT examination was 1.18 ± 0.11 (mean ± standard deviation, ranging from 0.86 to 1.29, with a mean contrast load of 97.7 mL and a mean weight of 84 kg ± 19.8, ranging from 60 to 130 kg.

### Bone mineral density assessment

**Fig. 2:**
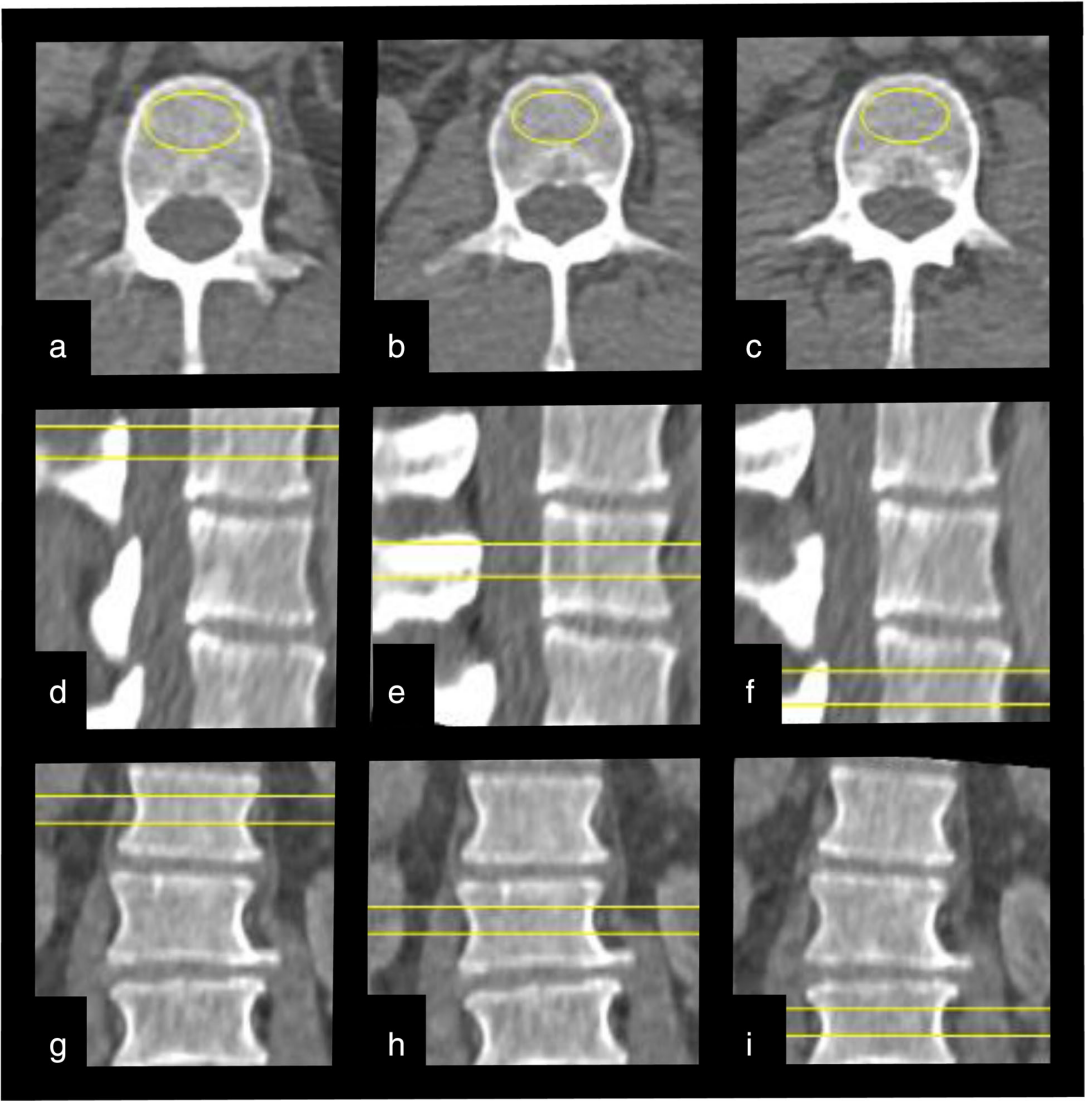
Images of the region of interest (ROI) placement in the Mindways software where panels **a**, **d**, and **g** represent vertebra L1 in the axial, sagital and coronal plane, respectively. Panels **b**, **e**, and **h** represent L2 in the 3 different planes while panels **c**, **f**, and **i** represent L3. The ROIs were made as large as possible, including as much trabecular bone material as possible, but carefully avoiding vessels, cortical, sclerotic, and cystic structures

For the vBMD analysis, Mindways qCT PRO software, version 2 (Mindways, Austin, TX, USA) was used as described in detail earlier [28]. In brief, preceding the study, calibration scans with Mindways dedicated phantom were performed, repeated monthly during the study period. After inclusion of patients, all scans were exported from the clinical picture archiving and communication system to a dedicated analysis computer. For the CTPI, we used every second scan for the vBMD analysis, resulting in a time resolution of 3 s for all time points (16 scans in the first phases and 3 scans in the later phase). The Mindways qCT PRO software uses semi-automatically placed regions of interest (ROIs) in the trabecular part of the lumbar vertebrae (middle part), which, if needed, could be manually adjusted before the vBMD values were calculated. The ROIs were made as large as possible, including as much trabecular bone material as possible, but carefully avoiding cortical, sclerotic, and cystic structures (Fig. 2). None of the patients had bone metastases, vertebral fractures or artefacts affecting bone analysis. The thickness of the ROI was 9 mm and vertebrae L1–L3 were included in the analysis, except for 2 patients in whom L3 was anatomically outside the scan length of the CTPI examination. The measurements were performed by an experienced osteoporosis physician (A.S.) with an over 15-year clinical experience. A vBMD < 80 mg/cm^3^ was considered as osteoporotic, a vBMD of 80–119 cm^3^ as osteopenic, and a vBMD > 120 mg/cm^3^ as normal regarding to the definition by the American College of Radiology [32].

### Radiation dose

The effective radiation dose was 20.9 mSv ± 3.6 (mean ± standard deviation), ranging from 16.0 to 30.4 mSv for CTPI and 22.5 mSv ± 7.9, ranging from 11.8 to 38.0 mSv for the 4-phase CT.

### Statistical analysis

SPSS Statistics (version 25.0, IBM Corp., Armonk, NY, USA) was used for the statistical analysis. As data showed normal distribution (tested with the Kolmogorov-Smirnov test and the Shapiro-Wilks test), parametric *t* test was used. Data is reported as mean ± standard deviation. For correlation analysis, Pearson correlation was used. A *p* value < 0.05 was considered significant.

## Results

### Descriptive

In total 43 vertebrae from 15 patients (11 males and 4 females) were included in the analysis. The age of patients was 71 ± 9 years (mean ± standard deviation), ranging from 55 to 86 years. Thirty percent of the vertebrae showed a vBMD < 80 mg/cm^3^, 63% a vBMD of 80–119 cm^3^ and 7% a vBMD > 120 mg/cm^3^. The mean weight of the patients was 84 ± 19.8 kg (mean ± standard deviation), ranging from 60 to 130 kg, and BMI was 27 ± 6 kg/m^2^, ranging from 20 to 42. Regarding renal function, mean creatinine was 80 ± 15 μmol/L, ranging from 59 to 112 μmol/L and an estimated glomerular filtration rate of 79 ± 20 mL/min, ranging from 43 to 123 mL/min.

### CTPI

**Fig. 3:**
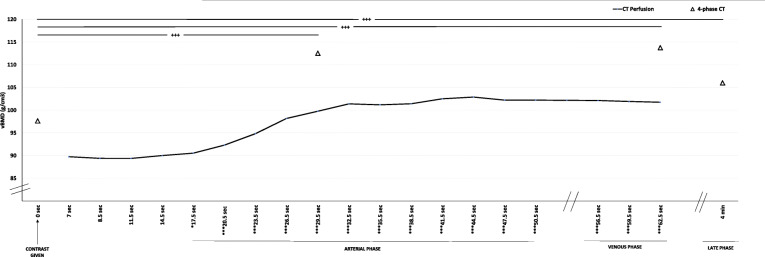
Volume bone mineral density (vBMD) over time in computed tomography (CT) perfusion imaging (CTPI) examination (black line) and 4-phase CT examination (triangles). In CTPI, statistically higher vBMD values were found for all time-points after 17.5 s (* in label of *x*-axis) compared to reference value at 7 s (unenhanced phase in vertebrae). Similar significant differences were observed for 4-phase CT where the arterial, venous, and late venous phase had significantly higher vBMD estimations than the unenhanced phase (+). */+ indicates *p* < 0.05, **/++ indicates *p* < 0.01, and ***/+++ indicates *p* < 0.001

**Fig. 4:**
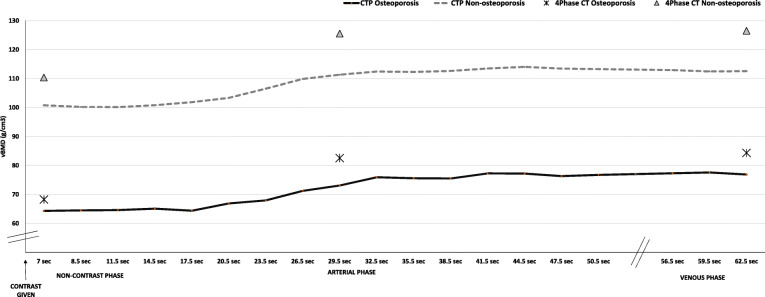
Volume bone mineral density (vBMD) value of osteoporotic (black line) and non-osteoporotic (dotted line) vertebrae in computed tomography (CT) perfusion imaging over time as well as vBMD values from osteoporotic (stars) and non-osteoporotic (triangles) vertebrae in 4-phase CT over time.

As shown in Fig. 2, vertebral vBMD showed a near-horizontal line until 17.5 s after contrast injection (*i.e.*, unenhanced phase in vertebrae), where after a steep increase in estimated vBMD, a plateau after around 41.5 s was seen. Compared to the first scan (unenhanced), the estimated vBMD values were significantly higher at all time points after 17.5 s, *i.e.*, in arterial and venous phases (Fig. 3). Volume BMD did not differ between two neighboring time points in the early phase (up to 17.5 s) and during venous phase (56.5−62.5 s). During the arterial phase, each time point differed significantly from the previous. There was no visual difference in the shape of the curves between osteoporotic and non-osteoporotic vertebrae (Fig. 4). Similarly, the two groups showed no significant difference in any time point regarding vBMD increase from first scan.

### Four-phase CT

As shown in Fig. 3, the 4-phase CT showed a similar pattern as CTPI, *i.e.*, significantly elevated vBMD in arterial (30 s) and venous phase (63 s) as compared to unenhanced scan.

The absolute vBMD change (delta) from non-contrast to arterial and venous phase, respectively, did not differ between osteoporotic and non-osteoporotic vertebrae (vBMD increase to arterial phase 14 + 5 mg/cm^3^ (osteoporotic) *versus* 15 + 6 mg/cm^3^ (non-osteoporotic), *p* = 0.645 and venous phase 16 + 4 mg/cm^3^ (osteoporotic) *versus* 16 + 7 mg/cm^3^ (non-osteoporotic), *p* = 0.974).

### Contrast load

A higher contrast dose/kg correlated to a higher vBMD both in arterial phase (*r* = 0.39, *p* = 0.001) and venous phase (*r* = 0.38, *p* = 0.001). As shown in Table 1, there were significantly higher vBMD increases in the 4-phase CT than corresponding time points in CTPI both regarding the arterial and venous phases. As described in the “Methods” section, 4-phase CT had nearly double the concentration of contrast/bodyweight than CTPI, *i.e.*, 1.18 ± 0.11 *versus* 0.62 ± 0.14.

**Table 1:** Change in vBMD after contrast administration

	CT perfusion	4-phase CT	***p*** value (paired)
**vBMD increase**^a^			
ARTERIAL	10.0 + 6.2	14.9 + 5.7	0.001
VENOUS	12.0 + 5.1	16.1 + 6.6	0.002

^a^Increase compared to non-contrast phase. *ARTERIAL* = 30 s and *VENOUS* = 63 s post-contrast injection

### Mathematical description of the vBMD curve

**Fig. 5:**
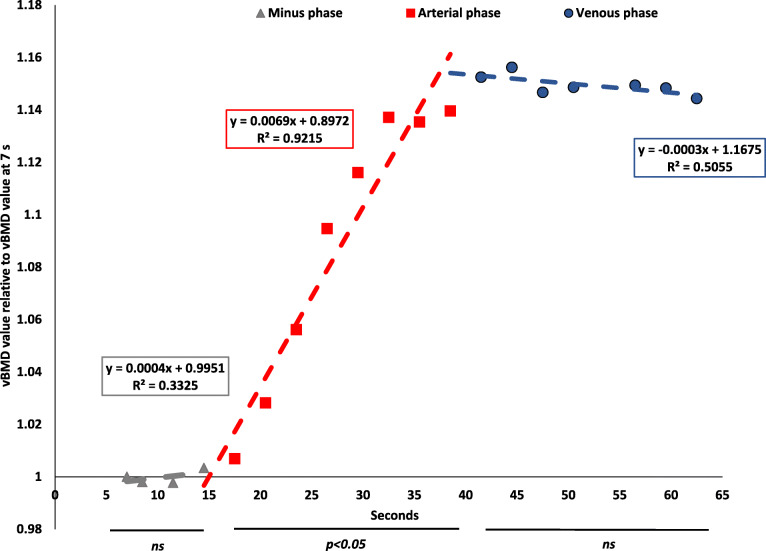
Normalised mean data of the relative change in volume bone mineral density (vBMD) compared to the vBMD value at 7 s (unenhanced phase in vertebrae) in the computed tomography perfusion imaging examination. The curve was divided in three subareas (unenhanced phase, grey; arterial phase, red; and venous phase, blue). As it can be seen visually and by the accompanying mathematical formula, the unenhanced and venous phase shows a near horizontal line while the arterial phase shows a steep increase of vBMD change over time. There was a significant change in neighboring time points between 17.5 s to 41.5 s. After that time point, there was no detectable difference between the neighboring time points

In Fig. 5, the CTPI graph (normalised vBMD values against 7 s) is divided in three main areas (non-contrast [grey], arterial [red], and venous [blue] contrast phase). The linear trend lines of the different areas show a steep increase during the arterial phase. In this section, all vBMD values differed significantly regarding vBMD in comparison to the previous time point. The non-contrast and venous phases show near horizontal lines and no difference between nearby scan.

## Discussion

We show that vBMD differs considerably within short timespans during the arterial phase, making potential correction formulas vulnerable as a short time shift in the CT protocol could cause significant overestimation or underestimation of the vBMD. In venous phase, vBMD behaviour is more stable, thus possibly enabling the use of correction formulas. Furthermore, the amount of given contrast agent (keeping the injection speed at the same rate) significantly affected the estimated vBMD at the corresponding time points. There was no difference in contrast uptake behaviour between osteoporotic and non-osteoporotic vertebrae. Thus, an estimated unenhanced vBMD value might be calculated from contrast-enhanced scans after the contrast has reached the time point about 41.5 s but with the caveat of taking the given amount of contrast and the contrast to weight ratio into consideration.

Osteoporotic screening based on CT scans [13, 15, 16, 22, 23, 25, 33–35] is of increasing interest as it might increase the number of osteoporotic patients being diagnosed and treated correctly as well as reducing the need of an additional examination for the patient. Early treatment of this group is important as the risk of new fractures is high shortly after a previous imminent fracture [6]. The screening approach was initially made by evaluating unenhanced abdominal CT scans for vBMD with a fixed kVp of 120 [15]. However, as an increasing number of abdominal CT scans are exclusively made after the administration of intravenous contrast agent in order to decrease radiation dose, several research groups have suggested to measure vBMD in the obtained contrast scans and correct the values by applying linear conversion functions [13, 15, 16, 22, 23, 25, 33–35]. However, our data, which is in line with the report by Acu et al. [27], indicate that the vertebral contrast load shows a nonlinear vBMD change over time, so that simple conversion formulas might not be applicable specifically in arterial phase scans [36]. The arterial phase is specifically important as the timing of contrast phases differs significantly between institutions and protocols. Our study shows that the vBMD values enter a plateau phase after 41.5 s reaching over the whole venous phase. This is in concordance with earlier studies [37] and it seems reasonable that a transformation formula might be described for these time points. The mathematical description given in Fig. 5 suggests that linear trendlines might be calculated for different contrast phases. These trendlines show that a small time-difference in the arterial phase (17.5–41.5 s) leading to significant differences in the vBMD estimation. Therefore, the use of such mathematical estimation trendlines should be avoided. However, in the venous phase (41.5–65 s), the trendline is more horizontal, leading to comparable vBMD values over time and therefore might be used to estimate vBMD values from venous scans.

As differences in the amount of given contrast and by that the contrast to weight ratio showed significant differences in the resulting vBMD values at every time point, it is important to also consider the contrast to weight ratio when calculating vBMD values. As the calculation of the vBMD values is based on the density measurement in HU, this finding is expected and in concordance to a study on contrast media behaviour in CT scans from Bae [30]. As shown by Roski et al. [24], the variability of contrast load and timing variances in the arterial phase might eventually be overcome by measuring the individual vessel iodine concentrations and by that adjusting the vBMD value.

Another important aspect when considering vBMD measurement is to be aware of the kVp used in the CT examination. A lower kVp yields exponentially higher HU values [29, 30], and furthermore impact of kVp varies between different tissues [28, 29]. For this reason, vBMD analysis with software methods based on internal tissue references, *i.e.*, no calibration phantom, might be problematic to use for kVp values other than 120. Phantom-calibrated software seems more plausible to use, but it is essential to have a calibration protocol for different kVp values. This limitation might be overcome by using dual-energy or photon-counting CT examinations, as the information from two different energies enables the calculation of vBMD without the need of fixed kVp values [38, 39].

Our study has several limitations. The sample size is small and the design is retrospective. As the study design was unique in the sense that both a CTPI and a 4-phase CT examination were performed under the same patient visit, the study size was limited by the ethics committee from the beginning. The study population was not a typical osteoporotic cohort in fracture liaison services, but do reflect and cover the range from osteoporotic to non-osteoporotic bone. However, as the primary outcome was not to describe the detection of osteoporotic patients, but to study the vBMD behaviour over time after contrast administration, the choice of study population seems to be adequate. Another limitation is the limited waiting time (10 min) between the CTPI examination and the 4-phase CT, resulting in remaining contrast agent in the scans of the 4-phase CT. To adjust for this, no statistical analysis was performed between the real vBMD values, but on their difference of vBMD over time. The ROIs were placed manually, which contributes to the risk of a higher intraobserver and interobserver variability. Finally, we did not correct for body weight in the study which might have impact on vBMD measurements in underweight or obese patients.

In conclusion, both time from contrast administration and contrast dose per kg affected vBMD results. In arterial phase, the steepness of the curve makes vBMD estimation unsure and questionable. However, as values plateaued after 41.5 s (early venous phase), it might be possible to predict the correct vBMD values from linear conversion formulas after that time point. This, however, requires the use of kVp calibrated analysis systems and measurements should be done with a constant contrast to weight ratio.

## Data Availability

The datasets used and/or analysed during the current study are available from the corresponding author on reasonable request.

## Abbreviations

CT: Computed tomography
HU: Hounsfield units
ROI: Region of interest
vBMD: Volume bone mineral density
